# Simultaneous Quantification of Dexpanthenol and Resorcinol from Hair Care Formulation Using Liquid Chromatography: Method Development and Validation

**DOI:** 10.1155/2016/1537952

**Published:** 2016-03-03

**Authors:** Amit Kumar De, Partha Pratim Chowdhury, Shyamaprasad Chattapadhyay

**Affiliations:** R&D Division, Dey's Medical Stores (Mfg.) Ltd., 62 Bondel Road, Kolkata, West Bengal 700019, India

## Abstract

The current study presents the simultaneous quantification of dexpanthenol and resorcinol from marketed hair care formulation. Dexpanthenol is often present as an active ingredient in personal care products for its beautifying and invigorating properties and restorative and smoothing properties. On the other hand resorcinol is mainly prescribed for the treatment of seborrheic dermatitis of scalp. The toxic side effects of resorcinol limit its use in dermatological preparations. Therefore an accurate quantification technique for the simultaneous estimation of these two components can be helpful for the formulation industries for the accurate analysis of their product quality. In the current study a high performance liquid chromatographic technique has been developed using a C18 column and a mobile phase consisting of phosphate buffer of pH = 2.8 following a gradient elution. The mobile phase flow rate was 0.6 mL per minute and the detection wavelength was 210 nm for dexpanthenol and 280 nm for resorcinol. The linearity study was carried out using five solutions having concentrations ranging between 10.34 *μ*g·mL^−1^ and 82.69 *μ*g·mL^−1^ (*r* ^2^ = 0.999) for resorcinol and 10.44 *μ*g·mL^−1^ and 83.50 *μ*g·mL^−1^ (*r* ^2^ = 0.998) for dexpanthenol. The method has been validated as per ICH Q2(R1) guidelines. The ease of single step sample preparation, accuracy, and precision (intraday and interday) study presents the method suitable for the simultaneous quantification of dexpanthenol and resorcinol from any personal care product and dermatological preparations containing these two ingredients.

## 1. Introduction

Dexpanthenol (DP) [D-panthenol or Provitamin B5] is the alcoholic analogue of D-pantothenic acid. Chemically it is 2,4-dihydroxy-N-(3-hydroxypropyl)-3,3-dimethyl-1-butanamide commonly present as an active ingredient in a number of Vitamin B-complex supplements and cosmetics (creams, ointments, lotions, etc.) for its beautifying and invigorating properties and restorative and smoothing properties [1–4]. DP is absorbed through the skin and gets converted into its active form, pantothenic acid (Vitamin B5), the precursor for the biosynthesis of coenzyme A. The acid form plays an important role in Krebs Cycle [4]. Pantothenic acid is also considered as “antistress vitamins” as its deficiency may result in different types of diseases like irritation of skin, dermatitis, depigmentation of the hair, and stunned growth [3, 5, 6]. In dermatological preparations it is usually given in strengths of 2 to 5%. Due to its restorative, smoothing properties and antidermatitis and depigmentation properties it is often used in hair care products [7] and some vitamin formulations.

The next candidate under our study is resorcinol (RC) or benzene-1,3-diol. It has got a number of pharmaceutical applications like treatment of acaine, seborrheic dermatitis, eczema, psoriasis, and other skin disorders. It is also used in hair coloring agents. However, this molecule has got a number of side effects on long term use or in large dosage [8, 9]. Research shows that a long term use of RC leads to reversible effects on human thyroid gland resulting in hypothyroidism [10, 11]. Therefore an exact method for the quantification of RC from formulations prescribed for medicinal and cosmetic use is essential.

Several cosmetic and pharmaceutical preparations contain both RC and DP either in form of excipients or as active components and very few methods have been reported for their quantification from such formulations. Again most of the reported techniques present methods for either RC or DP. Most of the methods described for the quantification of RC are spectrophotometric techniques [12, 13] and very few reported chromatographic techniques [14–17]. In another study conducted in our laboratory we developed a technique for the quantification of RC only from a marketed hair care product using an isocratic elution technique using liquid chromatography [18]. This method is however not suitable for the quantification of DP from such formulation which inspired us for the development of this newer technique. For DP only a single validated supercritical fluid chromatographic method has been reported [19]. The method describes the quantification of the active D-form in a racemic mixture of D and L forms of panthenol from cosmetic formulation using a mass spectroscopic detector. In the current study a simple, rapid, precise, and validated technique for the simultaneous quantification of both DP and RC from a marketed hair care product, in presence of a complex matrix comprising glycerin, ethanol, biotin, keratin hydrolysate, hyalkyl HBU, nicotinic acid, and polyvinylpyrrolidone, is presented.

## 2. Material and Methods

Dexpanthenol (DP) standard (purity 99.65%) and resorcinol (RC) standard (purity 99.98%) were purchased from Sigma Aldrich India Ltd. Hair care formulation containing DP and RC with batch number HV1124, manufacturing date February 2015, and expiry date July 2017 was used as a representative marketed formulation for study. Solvents of chromatography grade were purchased from Spectrochem India Limited. A C18 column 100 mm in length and 4.0 mm in internal diameter was purchased from Waters Limited (Waters MA, USA). All reagents used in analysis were purchased from Merck India Limited.

### 2.1. Equipment and Chromatographic Conditions

A Waters Alliance Separation Module (Waters, USA) quaternary gradient system and Waters 2489 dual lambda absorbance detector were used for the quantification purpose. The analysis was carried out using Empower 3 software (Waters, USA). A reverse phase Waters C18 column (Waters, USA) of 100 mm length, 4.0 mm internal diameter, 5 *μ*m particle size, and a gradient elution flow rate of 0.6 mL/min was used for analysis. The mobile phase was a combination of filtered and degassed 5.4 mM phosphate buffer (pH = 2.8) and acetonitrile (ACN) in proportions as presented in Table 1.

The analysis was carried out at ambient temperature and the injection volume was 20 *μ*L. The detection wavelength was 210 nm for DP and 280 nm for RC. Prior to chromatographic separation both the standard and the sample solutions were filtered through 0.2 *μ*m membrane filter (Pall Life science, India).

### 2.2. Preparation of Standard Solutions and Sample Solutions

#### 2.2.1. Standard Solution

**Figure 1:**
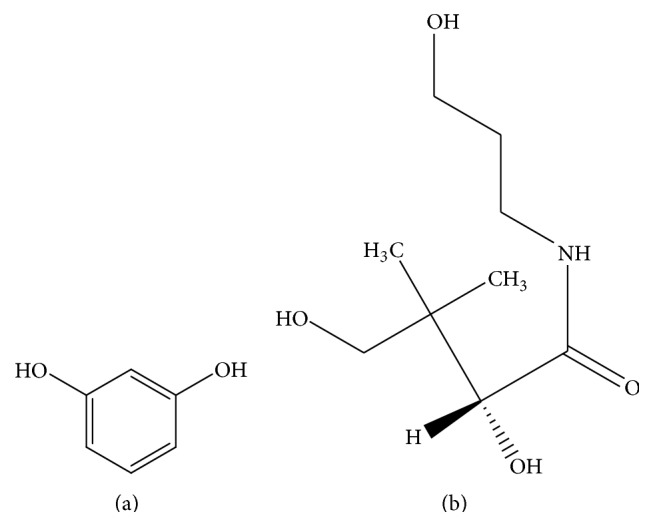
Chemical structure of resorcinol (a) and dexpanthenol (b).

The RC and DP stock solutions were prepared by dissolving 51.68 mg of RC (solution A) and 52.19 mg of DP (solution B) in 100 mL of diluents (90% buffer and 10% acetonitrile) in two separate clean and dry volumetric flasks using ultrasound. Solution A was diluted to the range 10.34 *μ*g·mL^−1^ to 82.69 *μ*g·mL^−1^ and solution B 10.44 *μ*g·mL^−1^ to 83.50 *μ*g·mL^−1^ using the diluents for analysis (Table 1). Peak area versus concentration curve was prepared (Figure 1(a) for RC and Figure 1(b) for DP) and was used for the determination of linearity of the method and the same was used for the quantification purpose. The results were presented in Table 2(a and b) for RC and DP, respectively. The curve fitting purpose was carried out following the least square method.

#### 2.2.2. Sample Solution

Preparation of sample solution was critical when a number of interfering substances were present within the matrix like glycerin, ethanol, biotin, keratin hydrolysate, hyalkyl HBU, nicotinic acid, and polyvinylpyrrolidone. However in our study, the method has been optimized following a simple single step sample preparation technique which was very much suitable for the regular analysis. 2 mL of the formulation was pipetted out accurately and transferred into a clean and dry volumetric flask and diluted to 25 mL with mobile phase. This solution was injected into the chromatographic system after filtering through 0.2 *μ*m membrane filter.

#### 2.2.3. Method Validation

**Figure 2:**
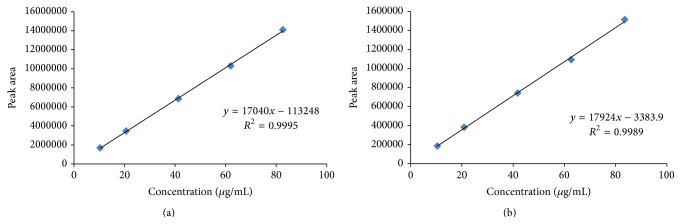
Concentration versus peak area curve for RC (a) and DP (b) analysis.

The high performance liquid chromatography technique for the simultaneous quantification of RC and DP was carried out following external calibration method [20, 21]. The analytical method was validated on the basis of accuracy, precision, linearity, range, and robustness of the method. The reliability and accuracy of the proposed method were determined on the basis of recovery studies. The sample solution was spiked with standard stock solution at three different levels (80%, 110%, and 120% of assay value for each of RC and DP labeled as RA, RB, and RC and DA, DB, and DC, resp.) [21]. Precession of the method was studied on the basis of precession of six replicate injections of the standard solution. The linearity was established through preparation of standard linearity curve in the concentration range of 10.34 *μ*g·mL^−1^ to 82.69 *μ*g·mL^−1^ for RC and 10.44 *μ*g·mL^−1^ to 83.50 *μ*g·mL^−1^ for DP (Figure 2). The same curves were used for quantification purpose. The intraday precision was calculated using six injections at the higher concentration range (41.34 *μ*g·mL^−1^ for RC and 41.75 *μ*g·mL^−1^ for DP) on the same day and on different days to obtain the interday precision. The peak purity study was used to determine specificity of the method. A photodiode array (PDA) detector was used for the purpose in place of UV-detector mentioned earlier, keeping the other chromatographic conditions unaltered. The limit of quantitation (LOQ) and the limit of detection (LOD) were determined following the technique described elsewhere [21]. Ruggedness of the method was determined by carrying out experiment on instruments of different make. Slight changes in the chromatographic conditions like composition (±5%) and pH (±0.1%) of mobile phase were made in order to study the robustness of the method.

The result obtained from each stage was subjected to statistical analysis based on Sigma plot software (Version 8.02 SPSS Inc., USA) and MS Excel 2007. The data were recorded in replicates and presented as mean ± standard deviation of the replicate measurements.

## 3. Results and Discussions

**Figure 3:**
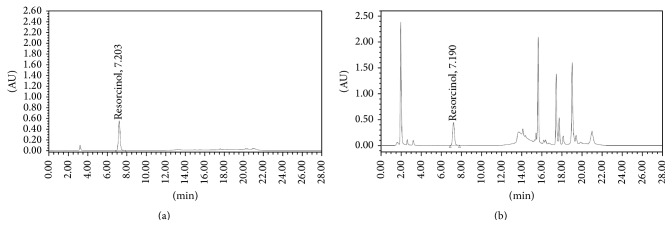
Representative chromatograms for resorcinol standard (a) and sample (b).

**Figure 4:**
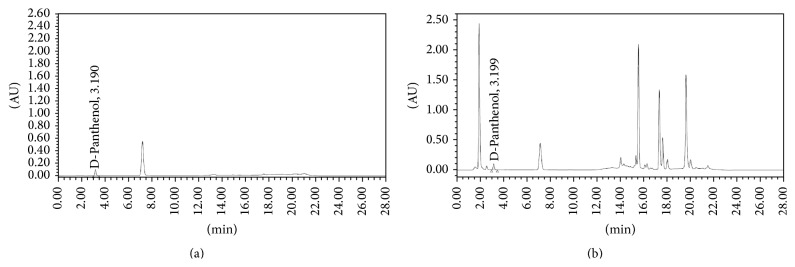
Representative chromatogram for dexpanthenol standard (a) and sample (b).

The average runtime for analysis was 28 minutes and the average retention time for RC was 7.20 ± 0.10 minutes and for DP was 3.198 ± 0.20 minutes and for RC and DP was 7.19 ± 0.10 minutes and 3.19 ± 0.10 minutes in sample solutions. Sufficient resolution was observed between RC and DP in the standard chromatogram and the sample chromatogram and the closely eluting peaks showed sufficient resolution in the sample chromatogram (Figures 3 and 4). This was probably the first reported technique for the simultaneous quantification of both from marketed hair care product. *λ*_max_ for RC was found at 280 nm and for DP at 210 nm. Therefore the analysis was carried out using a dual lambda absorbance detector with simultaneous scanning at 210 nm and 280 nm. The chromatographic purity for each component was analyzed using a PDA detector for each component under consideration. The peak purity angle for RC and DP was 0.129 and 0.217, respectively, and peak purity threshold was 0.337 and 0.411, respectively. Thus the analyte peaks were symmetrical, pure, and spectrally homogeneous and there is sufficient resolution between the respective analyte peaks in the sample chromatogram (Figures 3(b) and 4(b)).

### 3.1. Validation of the Developed Method

The quantification of RC was carried out on the basis of peak area and for DP on the basis of peak height. The method for analysis discussed earlier was validated as per USP and ICH Q2(R1) guidelines. The respective parameters for study were presented as follows.

#### 3.1.1. System Suitability

The suitability of the chromatographic system for performing the analysis was studied on the basis of system suitability. It was usually represented in terms of column efficiency, resolution, capacity factor, and tailing factor. The theoretical plates usually presented using the letter “*N*” define the column efficiency, a measure for peak sharpness, and were important for the detection of trace components. The peak resolution or resolution factor “*R*” determination was carried out to ensure that the closely eluting compounds were well separated from each other, to ensure the general resolving power of the system and also to ensure that the internal standards were well resolved for the analyte peak. The capacity factor “*k*” was a measure for the mass distribution of the analyte between the stationary and the mobile phase. The peak asymmetry was usually expressed in terms of symmetry factor or tailing factor. A value of unity was assigned for a perfectly symmetrical peak and the same increases with the increase in peak tailing. For a perfectly symmetrical peak its value was unity and the same increases as the tailing becomes more pronounced. The results were summarized as theoretical plates (*N* = 4.193282*e* ^+003^ for RC and 7.673464*e* ^+003^ for DP), capacity factor (*k* = 1.08 for RC and 1.32 for DP), peak asymmetry, or tailing factor (*t* = 0.97 for RC and 0.56 for DP) (Table 3) [20, 21].

#### 3.1.2. Linearity and Range

In order to study the linearity of the method, standard solutions in concentration range of 3.08 *μ*g·mL^−1^ to 24.67 *μ*g·mL^−1^ for RC and 10.44 *μ*g·mL^−1^ to 83.50 *μ*g·mL^−1^ for DP were used. The result obtained from the analysis was used to prepare the linearity curve. The curve thus obtained was found to be linear with regression factor *r* ^2^ = 0.999 and equation *y* = 17040*x* − 11324 for RC and *r* ^2^ = 0.998 and equation *y* = 17924*x* − 3383 for DP (Figure 2). The limit of detection (LOD) and limit of quantitation (LOQ) determined using the linearity curve [21] were 2.19 *μ*g·mL^−1^ and 6.64 *μ*g·mL^−1^ for RC, 0.62 *μ*g·mL^−1^ and 1.89 *μ*g·mL^−1^ for DP, respectively (Table 3). The results explained the sensitivity of the technique within the range of analysis under consideration [22].

#### 3.1.3. Accuracy

The accuracy was a measure of closeness of test results obtained by that procedure in comparison with the true value. It is necessary to establish the accuracy of the procedure in concentration range under consideration. In this study the accuracy was analyzed on the basis of recovery study. Three different solutions at concentration ranges 80%, 110%, and 120% of the calculated assay concentration were prepared for DP and RC, respectively. The solutions were prepared by adding a known volume of the standard solutions (solutions A and B) to the respective sample solutions. The results presented a mean recovery of 100.28% with % RSD 0.82 for RC and 100.02% and 0.48, respectively, for DP (Table 4). Therefore an appreciable accuracy was established over the range of study [21, 22].

#### 3.1.4. Precession

In our study precession was established on the basis of the standard deviation or relative standard deviation of a series of measurements like repeatability and intermediate precession of the developed technique. For repeatability study six sample solutions at 100% of test concentration were separately prepared and analyzed using the analytical procedure under study. The same sample solution was injected in triplicate for three consecutive days and the result thus obtained was used to determine the intermediate precession. The intraday precession presented a % RSD value of 0.19 and that for interday varied from 0.19% to 0.21% only for RC and 0.20 and 0.11 to 0.19 for DP making the analytical procedure precise under the range of study (Table 2).

#### 3.1.5. Specificity

The unequivocal assessment of the analyte under consideration from a mixture of components like impurities, degradation products, and matrix components was termed as specificity [21]. In this study specificity of both DP and RC was determined on the basis of chromatographic peak purity study of sample solutions. A peak was considered spectrally homogeneous, that is, free from coelution, only when the peak purity angle was less than threshold. The study presented the peak purity angle for RC and DP 0.129 and 0.217, respectively, and peak purity threshold 0.337 and 0.411, respectively (Table 3). Therefore it was concluded that the respective peaks were spectrally homogeneous, free from any coeluting impurities, and specific for the simultaneous analysis of both RC and DP.

#### 3.1.6. Robustness

The robustness was usually defined as the capacity of an analytical procedure to remain unaffected on introduction of small but deliberate variations of procedural parameters listed in the procedure documentation and present an indication of its stability during regular analysis. The robustness of the current method was studied on the basis of variations in analyst, instruments, and solution stability and on different storage conditions. The results were found within tolerance limits [20, 22] (Table 5).

The current method was found to be robust for the simultaneous quantification of RC and DP (Table 5) at concentrations as low as 6.64 *μ*g·mL^−1^ for RC and 1.89 *μ*g·mL^−1^ for DP (Table 2).

## 4. Conclusion

The reverse phase high performance liquid chromatographic technique developed in the current study was simple in terms of sample preparation and analysis, sensitive and selective for the quantification of both resorcinol and dexpanthenol simultaneously, and precise and accurate for their simultaneous quantification from a complex matrix. The method has been validated in terms of accuracy, precession, linearity, and sensitivity as per ICH Q2(R1) guidelines. The respective retention time for DP and RC was 3.19 minutes and 7.2 minutes. The peaks were well resolved from each other and other closely eluting peaks. Thus the current method is suitable for routine analysis, stability testing, and simultaneous quantification of RC and DP from various formulations available in the market.

## Figures and Tables

**Table 1:** Gradient elution composition.

Time (mins)	Flow rate	Solvent A (buffer pH = 2.8)	Solvent B (ACN)
0	0.6	90	10
8	0.6	90	10
18	0.6	10	90
19	0.6	90	10
28	0.6	90	10

**Table 2:** HPLC linearity.

(a) Resorcinol			(b) Dexpanthenol		
Concentration *µ*g·mL^−1^	Average peak area	% RSD	Concentration *µ*g·mL^−1^	Average peak area	% RSD
10.34	1693514	0.51	10.44	184197	0.95
20.67	3457115	0.11	20.88	381674	0.12
41.34	6854185	0.30	41.75	741349	0.21
62.02	10309345	0.42	62.63	1092022	0.38
82.69	14108460	0.56	83.50	1512696	0.44

**Table 3:** System suitability and method validation data for the simultaneous quantification of DP and RC.

Parameters	Resorcinol	Dexpanthenol
*System suitability*		
Retention time (min)	7.19	3.19
Capacity factor	1.08	1.32
Peak resolution	15.41^*∗*^	—
Tailing factor	0.97	0.56
Theoretical plates (*N*)	4.193282*e* ^+003^	7.673464*e* ^+003^
*Sensitivity*		
LOD (*µ*g·mL^−1^)	2.19	0.62
LOQ (*µ*g·mL^−1^)	6.64	1.89
*Precision*		
*Intraday*		
Mean content (mg/100 mL) (*n* = 5) ± % RSD	50.41 ± 0.19	49.77 ± 0.20
*Interday*		
Mean content (mg/100 mL) (day 1/day 2/day 3) (*n* = 3)	50.40/50.38/50.39	49.76/49.78/49.65
(% RSD) (day 1/day 2/day 3) (*n* = 3)	0.20/0.19/0.21	0.11/0.19/0.15
*Specificity*		
Peak purity angle	0.129	0.217
Peak purity threshold	0.337	0.411

^*∗*^With respect to DP.

**Table 4:** Results of accuracy study.

Solution	% of nominal value for RC	Concentration of final solution in *µ*g·mL^−1^	Recovery %	Observed concentration of final solution (*µ*g·mL^−1^)^*∗*^	Average assay value^*∗*^ (mg·100 mL^−1^)	Observed recovery %	Accuracy %	% RSD
	100	40.00	—	40.33	50.42	—	—	0.20
RA	80	32.00	80.00	32.27	40.35	80.03	100.03	
RB	110	44.03	110.07	44.93	56.17	111.40	101.21	0.82
RC	120	48.12	120.30	48.33	60.42	119.83	99.61	

Solution	% of nominal value for DP	Concentration of final solution in *µ*g·mL^−1^	Recovery %	Observed concentration of final solution (*µ*g·mL^−1^)^*∗*^	Average assay value^*∗*^ (mg·100 mL^−1^)	Observed recovery %	Accuracy %	% RSD

	100	40.00	—	39.82	49.77	—	—	0.02
DA	80	32.00	80.00	31.88	39.84	79.91	99.89	
DB	110	44.12	110.30	44.05	55.09	110.69	100.35	0.48
DC	120	47.99	119.97	47.69	59.61	119.77	99.83	

Note: ^*∗*^average from three replicate injections from sample preparations.

The recovery study is carried out over three-concentration range.

**Table 5:** Robustness study^*∗∗*^.

	Assay (mg/100 mL)	Analysts			Instruments			Storage condition		
		1	2	3	I	II	III	20°C	30°C	45°C
RC	50.42	50.41	50.40	50.44	50.41	50.42	50.42	50.42	50.40	50.40
DP	49.77	49.77	49.78	49.76	49.79	49.78	49.77	49.77	49.76	49.77

^*∗∗*^Three replicates each.
